# Phylogenetic analysis of a partial L1 gene from bovine papillomavirus type 1 isolated from naturally occurring papilloma cases in the northwestern region of Turkey

**DOI:** 10.4102/ojvr.v84i1.1450

**Published:** 2017-06-28

**Authors:** M. Ozkan Timurkan, M. Eray Alcigir

**Affiliations:** 1 Department of Virology, Atatürk University, Turkey; 2 Department of Pathology, Ankara University, Turkey

## Abstract

This study was aimed at the molecular characterisation of bovine papillomavirus type 1 (BPV-1) isolated from papilloma cases in the northwestern region of Turkey. BPV-1 is a widely occurring oncogenic virus in cattle and is associated with benign epithelial neoplasia which causes significant economic losses in dairy and beef cattle because of treatment costs. In this study, 29 suspected papilloma specimens were collected from cattle in northwestern Turkey. These samples underwent molecular characterisation via the polymerase chain reaction (PCR) and sequencing analysis as well as macroscopic and histopathological examination. The histopathological examinations confirmed papilloma as the main lesion type in the specimens. Of the 29 papilloma-like tissue samples that were collected, 11 (i.e. 37.93%) were detected as positive and determined as containing BPV-1 (11 of 11, 100%). Using a partial sequence for the L1 gene acquired from GenBank, phylogenetic analysis confirmed the presence of BPV-1 and revealed that the infection might have originated in cross bred domestic and imported cattle. This study provides potentially useful information on the origin and spread of this disease. Its results can potentially aid in the development of appropriate control measures and therapeutic or vaccination strategies against the BPV-1 strain of bovine papillomatosis.

## Introduction

Bovine papillomavirus (BPV) has a double-stranded, circular, 8-kb DNA genome. BPV displays tropism for mucosal and skin tissues, squamous epithelium and mesenchymal tissue. Infection is associated with the development of benign neoplastic lesions (Alcigir, Atalay Vural & Timurkan 2016; Shafti-Keramat et al. 2009). Although BPV types are species-specific, BPV-1, BPV-2 and BPV-13 can infect equids as well as cattle and cause the development of tumours in these species (Bocaneti et al. 2016). To date, 14 BPV types have been defined and classified into four genera: *Xipapillomavirus* (BPVs 3, 4, 6, 9, 10, 11 and 12), *Deltapapillomavirus* (BPVs 1, 2, 13 and 14), *Epsilonpapillomavirus* (BPVs 5 and 8) and *Dyoxipapillomavirus* (BPV 7) (Hamad et al. 2016; Lunardi et al. 2013).

Holstein, Simmental, Swiss and Jersey cattle, as well as each of these breeds crossbred with Anatolian Black cattle are the main cattle breeds in Turkey constituting important sources of dairy and beef products (Bor 2014). BPV genotypes cause papillomatosis, a disease which is responsible for significant economic losses because of growth reduction, weight loss and decreased milk production in the infected animals (Santos et al. 2016). Therefore, greater knowledge of this oncogenic disease would have a great impact on cattle breeding in Turkey. The natural carriers and primary source of BPV are cattle. The virus enters the body through scratches or other injuries, and infection spreads via both direct and indirect contact with infected cattle. The infection also appears to spread through contact with contaminated materials, milking machines and semen. Other factors, including malnutrition, hormonal imbalances, mutations and long-term exposure to sunlight can increase the risk of infection by causing immunodeficiency (Hamad et al. 2016; Lindsey et al. 2009).

Recent epidemiological reports indicate that BPV is prevalent in Asian countries such as Iraq (Hamad et al. 2016) and Turkey (Ataseven, Kanat & Ergun 2016; Dagalp et al. 2017); European countries such as Italy (Grindatto et al. 2015); African countries such as Nigeria (Jeremiah, Fagbohun & Babalola 2016) and American countries such as Brazil (Alcântara et al. 2015) and Mexico (Löhr et al. 2005).

This study aimed to identify and characterise the BPV-1 strains prevalent in Turkey. This study demonstrates the molecular detection and characterisation of BPV DNA from cutaneous warts in some indigenous breeds of cattle in northwestern Turkey.

## Materials and methods

### Animals and sample collection

Within this context, samples were collected from cases diagnosed as papilloma, a national project (Ankara University, Research Scientific Projects; Project no: 14B0239001), and sample collection continued after the project ended. Of all the samples from suspected papilloma cases, 29 were collected by registered veterinarians from several cattle slaughterhouses in various provinces (Ankara, Bursa and Samsun) of northwestern Turkey. Of the 29 samples, 11 were from Anatolian Black, Holstein, Simental and Jersey (half-breed) cattle with a papilloma diagnosis. The study sample also included cows with bovine papillomatosis that were brought to private clinics by their owners between March 2014 and November 2016 (Table 1). The collected samples had varying diameters (1 cm – 10 cm) and were harvested from different parts of the body (such as the udder and teat, neck, cranial and caudal abdominal region, and also plica genus). Each sample was immediately divided into two parts, which were either stored in a deep freezer for subsequent molecular analysis or fixed in 10% neutral buffered formalin for histologic examination (Hamad et al. 2016).

**TABLE 1:** Bovine papillomavirus samples and their origins.

Number	Sample ID	Origin (provinces)	Age (year)	Breed	Samples	Collection date	Accession number
1	TR-Ank-1	Ankara	3	Holstein half bred	Plica genus	06.03.2014	KY372394
2	TR-Ank-2	Ankara	3	Cross bred Anatolian Black	Cranial abdominal region and back skin	12.04.2014	KY372388
3	TR-Bur-1	Bursa	4	Simental half bred	Caudal abdominal region	27.09.2015	KY372392
4	TR-Bur-2	Bursa	1.5	Simental half bred	Udder	27.09.2015	KY372391
5	TR-Bur-3	Bursa	3	Holstein half bred	Udder and teat	03.10.2015	KY372390
6	TR-Sam-1	Samsun	3	Anatolian Black	Caudal abdominal region	04.11.2015	KY372393
7	TR-Sam-2	Samsun	2.5	Jersey half bred	Neck	04.11.2015	KY372395
8	TR-Sam-3	Samsun	4	Jersey half bred	Caudal abdominal region	17.11.2015	KY372396
9	TR-Sam-4	Samsun	3	Cross bred Jersey	Udder	01.02.2016	KY372389
10	TR-Sam-5	Samsun	3.5	Holstein half bred	Cranial abdominal region	09.05.2016	KY372387
11	TR-Sam-6	Samsun	3.5	Cross bred Holstein	Neck	14.09.2016	KY372386

### Polymerase chain reaction analysis

A nucleic acid extraction kit (Vivantis Technologies Sdn Bhd, Shah Alam, Selangor, Malaysia) was used to extract DNA from 11 frozen tissue samples according to the manufacturer’s instructions. A primer set specifically designed for BPV-1 was used (forward 5’ – GGAGCGCCTGCTAACTATAGGA – 3’; reverse 5’ – ATCTGTTGTTTGGGTGGTGAC – 3’) to obtain 301 bp DNA fragments. The samples were also evaluated in terms of BPV-2 and BPV-4 primer sets (Lindsey et al. 2009). The polymerase chain reaction (PCR) amplification was performed following a standard protocol in 100 mM Tris HCl (pH 9.0), 500 mM KCl, 1.5 mM MgCl_2_, 0.2 mM dNTP, 0.25 mM of each primer and 2.5 units of Taq DNA polymerase (Thermo Fisher Scientific, Waltham, MA, USA). The PCR cocktail (final reaction volume, 30 µL) was amplified under the following conditions: 35 cycles of denaturation at 94 °C for 60 s, annealing at 50 °C for 60 s and extension at 72 °C for 60 s followed by a final extension at 72 °C for 10 min. The PCR products were detected by electrophoresis on a 1.0% agarose gel containing ethidium bromide, which was placed in a Tris-acetate-EDTA (TAE) buffer and run at a constant voltage (120 V) for approximately 25 min. DNA was visualised using a Vilber Loumart Gel Documentation System (Vilber Lourmart, Marne-la-Vallée, France).

### Bovine papillomavirus type 1 sequencing

A total of 11 BPV-1-positive specimens, as confirmed by PCR, were selected as representative of the BPV’s geographical distribution and their viral genome type was sequenced. For sequencing analysis, PCR products were purified using a Genejet Gel Extraction Kit (Thermo Fisher Scientific, Waltham, MA, USA). The cleaned PCR products were sequenced using a Big Dye Terminator v3.1 cycle sequencing Kit (Applied bio systems, Forester City, CA, USA) at the Pendik Veterinary Control Institute, İstanbul, Turkey. Sequenced products were run on the Applied Biosystems ABI, 3100 (Applied Biosystems, Forester City, CA, USA).

### Phylogenetic analysis of bovine papillomavirus type 1

The sequences obtained in this study were subjected to phylogenetic analysis. Sequences were aligned using BioEdit software (version 5.0.6; North Carolina State University, Raleigh, NC, USA) (Hall 1999). To identify evolutionary relationships among the analysed sequences, we performed phylogenetic analysis with a maximum-likelihood phylogenetic tree by using Molecular Evolutionary Genetics Analysis (MEGA) software (version 6.0; Tokyo Metropolitan University) (Tamura et al. 2013). The maximum-likelihood phylogenetic tree was used to generate a general time-reversible model by using a discrete gamma distribution with five rate categories and test of phylogeny was performed using a bootstrap method with 1000 bootstrap replicates.

### Pathomorphological examination

Eleven BPV-1 positive cases, which included solid masses, were derived from various body parts including the neck (*n* = 2), plica genus (*n* = 1), cranial and caudal abdominal region (*n* = 5) and mammary region (*n* = 3). Papilloma suspected masses were evaluated according to size, weight, elasticity, colour and appearance of the outer and cut sections. All collected samples were processed routinely after fixation in 10% buffered formalin. The samples were embedded in paraffin wax and cut into 4-μm-thick sections. The sections were stained according to haematoxylin-eosin (H&E) staining protocols, then evaluated using a light microscope (Leica, DM4000) and photographed with a camera attachment (Bioscience, MBF CX9000).

## Results

### Polymerase chain reaction results

BPV-1 DNA was detected in 11 of the 29 collected papilloma samples. All 29 samples were negative for BPV-2 and BPV-4 DNA. The 11 BPV-1-positive samples produced DNA fragments for the L1 gene that were 301 bp in length. These fragments were amplified using BPV-1 specific primers. PCR analysis showed that 100% (11/11) of the analysed bovine papillomatoses were induced by BPV-1 in the assessed Turkish cattle populations which included both imported breeds and crossbreeds being raised in Turkey.

### Bovine papillomavirus type 1 sequencing

Sequencing analysis was conducted on the L1 gene PCR products amplified from the collected samples and revealed that they were similar and of a single type (BPV-1). The results confirmed the presence of BPV-1, with a 97% sequence identity to the majority of the Basic Local Alignment Search Tool (BLAST)-searched BPV-1 sequences. This finding is a genotypic confirmation of the presence of BPV-1 as a primary causative agent of bovine papillomatosis in cattle in Turkey.

### Evaluation of phylogenetic analysis

**FIGURE 1:**
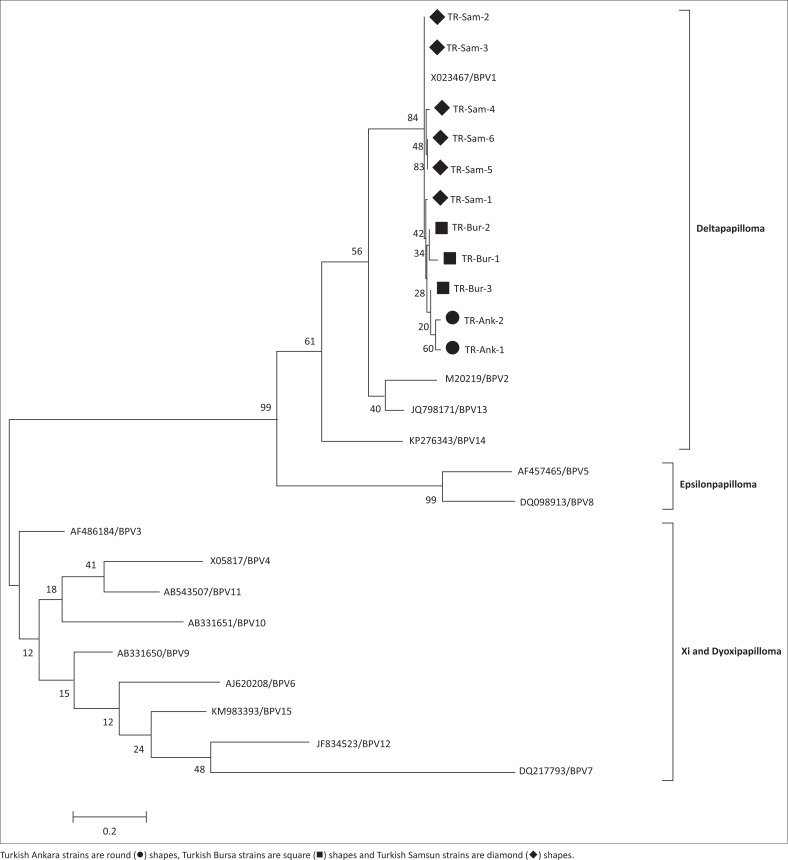
Phylogenetic tree showing the identified Turkish BPV sequence. The sequence was found to belong to the genus *Deltapapillomavirus*. The sequence was constructed via the maximum likelihood method using MEGA 6 software.

A phylogenetic tree was generated using retrieved genome sequences that were deposited in GenBank and compared with the sequence (Figure 1) of the 11 BPV Turkish strains (in Table 1 with accession numbers). The aligned sample sequences were classified as BPV-1 (genus *Deltapapillomavirus*). The aligned sequences showed a high percentage sequence identity to the nucleotide sequence of BPV-1 and to the sequence of a BPV strain isolated in Japan (Accession number: X02346).

### Pathomorphological findings

**FIGURE 2:**
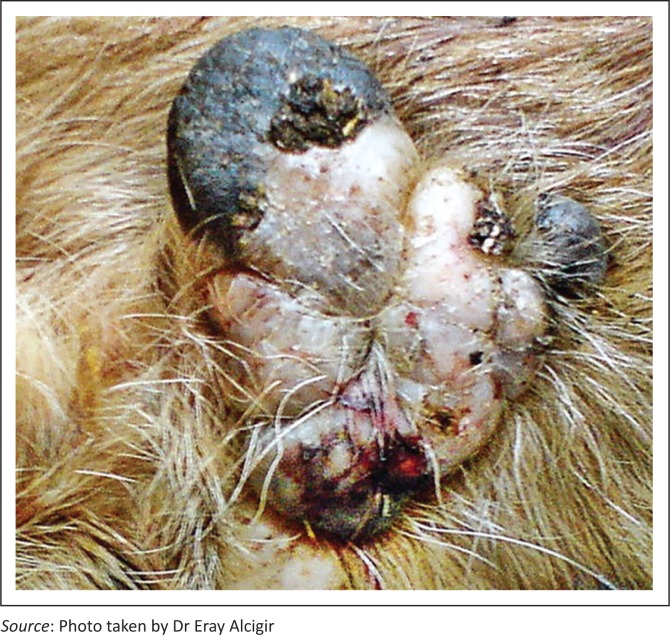
Macroscopic appearance of papilloma.

**FIGURE 3:**
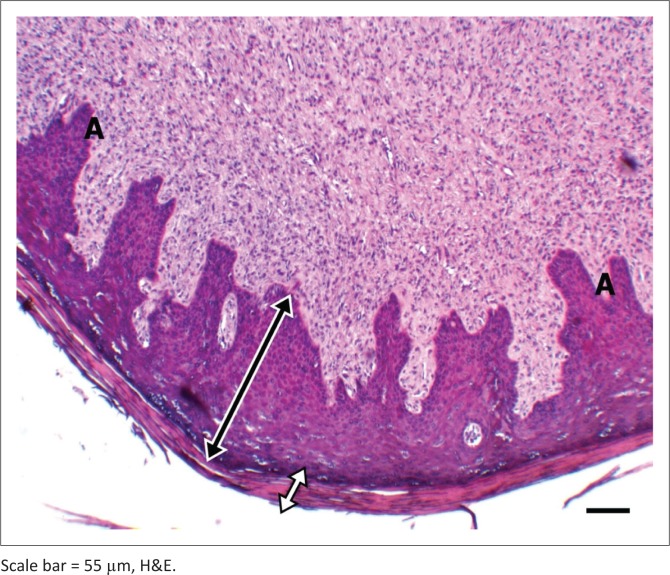
Spinosum layer proliferation (bilateral white arrows), hyperkeratosis (bilateral black arrows), acanthosis (A).

**FIGURE 4:**
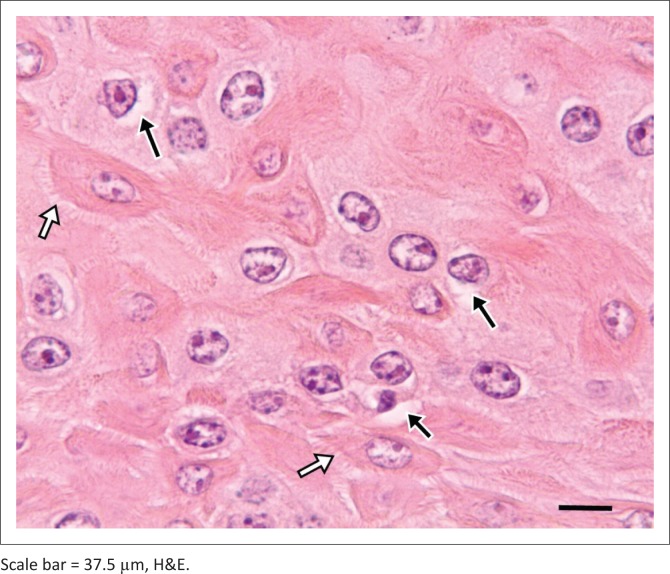
Vacuolar degeneration in spinosum layer cells (black arrows), formation of bridges between epithelial cells because of stretching of tonofibrillary junctions (white arrows).

Macroscopically resected skin samples suspected of papilloma were greyish-white multilobulated outgrowths with a firm consistency (Figure 2). Some of the skin showed brownish-black pigmentation. The cut sections of the skin warts were dark brownish-black. Homogeneous, greyish-white connective tissue was observed just beneath the warts. Histopathological analysis revealed that the epidermal part of the tumours comprised mostly hyperplastic spinosum layer cells and other proliferative neoplastic epidermal cells. Hydropic or vacuolar degeneration was observed in some hyperplastic spinosum cells, as well as koilocytes that were characterised by enlarged, pale eosinophilic and vacuolated cytoplasm, and pyknotic nuclei with eccentric localisation. This gave rise to a reticular appearance together with epithelial junctions in the form of *bridges*. In the granular layer, keratohyalin granules of eosinophilic and cytoplasmic particles were attached to those cells. In addition, acanthosis, orthokeratosis and parakeratotic hyperkeratosis were present (Figures 3 and 4). In addition, some proliferative activity and vascularization was observed in the connective tissue of papilloma consisting of parenchyma.

## Ethical considerations

This study was approved and confirmed by the Local Ethical Committee at Ankara University, Ankara, Turkey (number: 53184147/56050, date: 21 October 2013).

## Discussion

In this study, PCR was used to analyse viral DNA and evaluated histopathology diagnosed cases of bovine papillomatosis to identify and characterise the BPV-1 genotype that exists in Turkey and to detect its lesions on cattle skin. Genotype-specific primers were used to identify and characterise BPV-1 and findings were confirmed by sequencing and phylogenetic analysis, which are considered to be the most appropriate methods for characterising BPV (Grindatto et al. 2015).

Several studies (Grindatto et al. 2015; Santos et al. 2016) have been conducted to determine the prevalence of BPV infection in cattle, the most prevalent types of the virus, their geographic distributions and the risk factors involved in the development of skin papillomas. Some recent Turkish studies (Ataseven et al. 2016; Tan et al. 2012) described PCR-based molecular typing of BPVs in cattle with cutaneous papillomatosis, but this typing was not confirmed by phylogenetic analyses. In our study, we concluded that infection with several of the BPV types described in the aforementioned studies does not always cause papillomas. Sometimes, fibropapilloma and papilloma-like lesions can also occur on the skin of cattle. However, in this study, we found a greater tendency for papilloma development among warts on cattle skin infected with several sub-clusters of BPV-1 with this finding supporting a papilloma diagnosis. This situation was of interest because different BPV-1 strains with a wide geographical distribution are found in Turkey. Our results indicate that most BPV infections in cattle are caused by BPV with only one genotype. In contrast, other studies have revealed the occurrence of coinfections (Grindatto et al. 2015), some with two, three and four BPV genotypes having been reported (Lindsey et al. 2009). However, we detected only BPV-1 in our study. This finding might indicate the resistance of the animals to other genotypes, or it may suggest that animals are infected by BPV-1 only once in their lives.

BPV-1 DNA was detected in 11 samples in our molecular analyses, confirming the presence of BPV-1, which belongs to the genus *Deltapapillomavirus*. Phylogenetic analysis showed that the strains were closely clustered according to province. Furthermore, the Samsun strains, which are part of the same sub-cluster, were closer to the reference strain (Accession number: X023467) but the Ankara and Bursa strains were not. This study revealed that highly pathogenic BPV-1 is widespread in Turkey, especially in the cities of Ankara, Bursa and Samsun. This genotype has been found in association with the development of cutaneous papillomatosis as papilloma or fibropapilloma (Alcigir et al. 2016). This genotype (BPV-1) has been found in association with the development of cutaneous papillomatosis manifesting as papilloma or fibropapilloma (Alcigir et al. 2016). When the strains in our study were grouped together, we discovered two different sub-clusters, namely the Ankara strains and the Samsun–Bursa strains, with each sub-cluster being closely related to each other and similar in terms of nucleotide sequences. We also found some chronological differences and similarities among the collected samples. For instance, the samples from the Ankara province were collected in 2014, whereas the samples from the Samsun and Bursa provinces were collected in 2015 and 2016.

Amplicons obtained from BPV isolates via PCR were sequenced and comparative sequence analysis showed a 93.9% – 100% identity among the BPV isolates and 95.0% – 99.2% identity between our isolates and the BPV-1 reference strain (X023467) (data not shown). In addition, other papillomaviruses tend to cause papillomas in organs such as the bladder and more frequently, in the oesophagus. These lesions occur more commonly with the solid forms. Therefore, it appears likely that that BPV-1 might have an affinity for the skin, resulting in lesions on the skin rather than on the mucosa.

**FIGURE 5:**
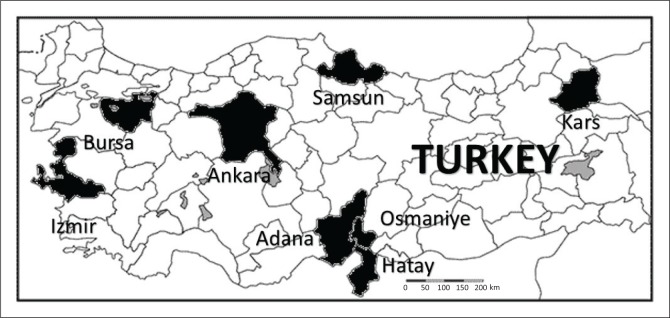
Map of Turkey showing location of the studies in black.

To the best of our knowledge, this is the first molecular characterisation of BPV-1 according to geographic distribution in Turkey since previous studies did not investigate the presence of BPV-1 in the Samsun and Bursa provinces. In the current study, we showed that BPV-1 infection is also prevalent in the Ankara province, a finding that contributes significantly to the body of knowledge regarding the geographical distribution of the various types of BPV in Turkey because a limited number of molecular studies were previously conducted in this region, such as those of Dagalp et al. (2017) in the provinces of Ankara, Hatay, Adana, Izmir and Kars and by Ataseven et al. (2016) in Adana, Hatay and Osmaniye (Figure 5). We propose that conducting more phylogenetic studies will further our understanding of genetic relationships among the BPV types and their relative prevalence across the different provinces in Turkey. Identifying these strains and developing regional vaccine programmes are extremely important.

Analysis of BPV-1 isolates collected from the northwestern provinces of Turkey revealed a possible relationship with papilloma lesions as indicated by the present study results, or with fibropapilloma lesions as indicated by the results of Alcigir et al. (2016). However, further study is warranted to determine the relationship between specific neoplastic lesions and the corresponding strains of BPV-1. In terms of both gross pathology and histopathological findings, this close relationship was present between multiple formations, shapes and epidermal and/or connective tissue patterns of neoplastic tissue in papilloma cases from older cattle. We observed that papilloma masses were mostly encountered in the thin-skinned regions of young cattle. Further analysis of statistical data is required to elucidate this close relationship. The results of this study suggest that BPV-1 infections may occur preferentially in thin-skinned areas or after corruption of skin integrity because of trauma such as abrasions in susceptible regions such as the plica genus, abdominal region, udder and teats. Therefore, those locations may have a greater predisposition to viral infections in young cattle. Accordingly, BPV-1 cutaneous oncogenicity should be further investigated on the basis of the localisation of tumours, cattle age and breed. In addition, it must be determined whether these predispositions are characteristic of Anatolian Black, Holstein (half-breed), Jersey (half-breed) as well as other cattle breeds. The papillomavirus, especially BPV-1, might have the ability to evolve between Anatolian crossbred herds. However, new studies in this field have enabled the elucidation of molecular and histological differences among the subtypes or strains of the different types of BPV.

## Conclusion

In conclusion, to our knowledge, this is the first study to detect and characterise the BPV-1 genotype in Turkey. Its findings have assisted in detecting BPV in neoplastic tissue by clarifying its interaction with the skin. In the longer term, our findings may also help to reduce the economic losses related to bovine papillomatosis by providing the basis for the implementation of vaccination programmes in northwestern Turkey.
